# Editorial for the Special Issue “Molecular Biology in Targeted Radionuclide Therapy Radiopharmaceutical Design”

**DOI:** 10.3390/cimb46030152

**Published:** 2024-03-13

**Authors:** Carlo Aprile, Onelio Geatti, Letizia Canziani, Lorenzo Lodola

**Affiliations:** 1 Fondazione IRCCS Policlinico San Matteo, 27100 Pavia, Italy; l.canziani@smatteo.pv.it; 2 Studio di Radiologia Bazzocchi, 34135 Trieste, Italy; ogeatti@gmail.com

## 1. Introduction

Targeted radionuclide therapy (TRT) is gaining wide and rapid acceptance in clinical practice as it can deliver alpha or beta irradiation to a tumor-associated target which may be present in the tumor cell itself or in the microenvironment. The challenges related to this approach involve many biological aspects, such as determining the most reliable cellular or interstitial target and the most suitable vector able to carry the nuclide to the target, avoiding damage as much as possible with minimal detrimental effects on normal tissues. This Special Issue focuses on four selected articles, briefly described in the following paragraphs, which describe innovative scientific insights into many aspects of the molecular mechanisms related to TRT.

## 2. An Overview of Published Articles 

1. Most differentiated thyroid cancer (DTC) patients have an excellent prognosis, but local recurrence and distant metastases occur in up to 20% and 10% of cases, respectively. However, about one-third of DTC patients with recurrent or metastatic disease lose the hallmark of specific iodine uptake initially or gradually and acquire radioactive iodine-refractory DTC (RAIR-DTC) caused by the decreased expression of sodium iodide symporter (NIS). The prognosis patients is poor for these patients, a 10-year survival rate <10%. Point mutations in the RTK/BRAF/MAPK/ERK and PI3K-AKT-mTOR pathways, chromosome rearrangement, or aberrant gene methylation are thought to be responsible for diminished NIS signaling. In particular, BRAF ^V600E^ mutations are inversely related to NIS expression. Tyrosine kinase inhibitors (TKIs) have been demonstrated to significantly improve progression-free survival; however, several drawbacks are associated with their long-term administration. Full insight into the molecular mechanisms of RAIR-DTC can lead to the development of new drugs able to improve survival [1,2,3,4]. In this Special Issue, Lee and coworkers [contribution 1] used clinical and molecular data from the dataset of The Cancer Genome Atlas for Thyroid Cancer (TCGA-THCA) to explore potential pathways associated with the loss of NIS expression. They found that NIS expression is negatively correlated with tumor size, and a low level of expression is associated with recurrence-free survival. Through a propensity-score-matched analysis, their transcriptome analysis identified several novel pathways that could serve as potential targets in future studies to reverse the loss of NIS expression After matching for clinicopathologic profiles and driver mutations, a principal component analysis revealed distinct gene expressions between high- and low-NIS groups, identifying several potential targets.

2. A radiation injury due to external beam or radionuclide therapy or accidental exposure is able to affect many cellular pathways, producing heterogeneous effects across exposed subjects. Frequently, physical dosimetry is not able to assess the true absorbed dose, which can be different from the extent of biological perturbation; therefore, there is a need for reliable biomarkers able to correlate exposure and biological system changes. Biodosimetry examines changes induced by IR in chromosomes, metabolomics, proteomics and other molecular processes. Seven fields of biodosimetry have been identified: cytogenetics, electron paramagnetic resonance, proteomics, metabolomics, genomics, lymphocyte kinetics and transcriptomics. These biomarkers might be able to aid in the prognosis and early treatment of normal tissue damage, validating the dose exposure and assessing long-term effects after exposure. Among these fields, the proteomics method is the least studied, although it can be a promising and powerful tool for discovering new biomarkers [5,6,7]. In this Special Issue, Alkhalil and coworkers [contribution 2], working from different research centers in US, address a very important and topical issue to gain detailed insight into the effects of ionizing radiation (IR) on the skin and search for biomarkers with biodosimetry applications. Mouse skin biopsies at various times after exposure to whole-body ionizing radiation were evaluated for the potential application of transcriptional alterations in radiation diagnosis and prognosis. The numbers of SDTGs (significantly differentially transcribed genes) and the percentages of upregulated SDTGs revealed stationary downregulation post lethal dose in contrast to responses to sublethal doses, which were dynamic and largely upregulated. The focus of this report was to introduce indicative transcriptomic patterns and describe their potential applications in radiation exposure.

3. Theragnostics is a new approach that combines diagnostic imaging and radionuclide therapy. It is based on the use of a pair of nuclides, one to be used in PET or SPECT imaging and the other (an alpha or beta minus nuclide) to exert therapeutic effects. The use of theragnostic pairs has increased considerably in recent years, not only to select patients who are eligible for TRT but also to calculate an efficacious dose for hitting the target without damaging normal tissues. The expression of SSTR (Somato Statin Receptor) by NETs (neuro-endocrine tumors) offers a very specific target for diagnostic imaging and therapy. The search for a more efficient theranostic candidate must take into account several parameters, including (1) the peptide sequence/chelator and its affinity for receptor subtypes; (2) the characteristics of the isotope and its SPECT/PET suitability [8,9,10,11,12]. Poletto and coworkers [contribution 3] performed a systematic review of the existing literature in this field. The rates of true positivity were 63.7%, 58.5%, 78.4% and 82.4%, respectively, for 111In-DTPA-Octreotide, 99mTc EDDA/HYNIC-TOC, 68Ga-DOTATATE/TOC and 64Cu-DOTATATE. As expected, PET tracers appear more suitable for the in vivo identification of NETs. Among the radiopharmaceuticals taken into account, 64Cu-labelled peptides seem to offer better spatial resolution in comparison to 68Ga-labelled ones and, in addition, they might represent true theragnostic agents after therapeutic 67Cu labelling. Interestingly, the use of an antagonist instead of an agonist seems to offer more favorable pharmacokinetics and image contrast and greater tumor uptake with longer residence time. 

4. The design of a radiopharmaceutical to deliver therapeutic radionuclides to tumor tissue is still an open challenge and requires choice to combine a suitable vector with the appropriate nuclide. Among the different factors to be taken into account, the following are likely the most relevant: (a) the type and location of the biological target and (b) the time constraints imposed by the nuclide’s half-life. For instance, the identification of possible alternatives to the direct labeling of MAbs and peptides may improve the delivery of radionuclides to the tumoral target, ensuring better therapeutic efficacy without a detrimental effect on normal tissues [13,14]. In a systemic review, Poletto et al. [Contribution 4] from Padua University analyzed three possible delivery strategies in tumor treatment: (1) the labeling of radionuclides on liposomes, (2) a pre-targeting strategy based on the avidin–biotin interaction and (3) the feasibility of designing new ligands with greater affinity for their receptors by virtually simulating their interactions (docking) with the receptors. All strategies offer therapeutic potential but also have several drawbacks. It is quite difficult to identify the best approach for several reasons, including the small number of patients and their heterogeneity. The authors conclude that the most promising option is probably the one based on the use of new ligands identified via docking, that is, the prediction of ligand–target interactions, knowing the three-dimensional structure of the target. In this regard, the use of computer modeling and simulation, referred to as “in silico medicine”, can predict outcomes for many variables, such as target-binding properties and pharmacokinetics. These approaches highlight the additional value of integrating Artificial Intelligence into radiopharmaceutical design, revolutionizing precision medicine [15].

## 3. Conclusions

Advancements in the field of precision medicine have changed the landscape of cancer treatment. Improved radiopharmaceutical design and delivery, along with deeper insights into the biochemical and genetic mechanisms governing tumor progression, allow for tailored detection and disease stratification as well as therapy. Recently, radiopharmaceutical design has been defined as a “hot” topic and a rapidly evolving matter [16], and the contributions in this Special Issue encompass a wide range of research studies which elucidate the richness of this field.
